# Classification, Chemical, and Toxicological Properties of Carbamate Nerve Agents

**DOI:** 10.3390/jox14040092

**Published:** 2024-11-07

**Authors:** Georgios Pampalakis

**Affiliations:** Laboratory of Pharmacology, School of Pharmacy, Aristotle University of Thessaloniki, 54124 Thessaloniki, Greece; gpampalakis@pharm.auth.gr

**Keywords:** nerve agents, chemical warfare, carbamates, quaternized ammonium salts

## Abstract

Nerve agents are usually identified as exceedingly toxic organophosphate compounds like VX and sarin. Nevertheless, although carbamate nerve agents (CNAs) have been developed they constitute the least studied class of nerve agents outside military literature. Recently, CNAs gained popularity after the inclusion of a small subset of carbamate agents in the Chemical Weapons Convention (CWC) list of Schedule 1 chemicals. Here, a holistic approach was used to identify and categorize the developed CNAs. It is demonstrated that CNAs encompass a highly diverse class of compounds. Their main characteristics include an aromatic group that carries the carbamate moiety. Based on their chemical structure, CNAs were categorized into two generations that are further divided into various subclasses. The second generation of CNAs includes some members that exhibit higher toxicity than VX. CNAs have not been used in any conflict, which may be related to their solid nature that requires sophisticated delivery systems. Since, however, CNAs can be applied as poisons in individualized cases, understanding their chemistry and toxicological properties is important for the development of effective countermeasures.

## 1. Introduction

Nerve agents are known as organophosphate chemicals that inhibit acetylcholinesterase (AChE) and are classified as G-, V-, and A-agents (Novichok) [1,2,3]. Nevertheless, there is another significantly less studied and discussed class of nerve agents, carbamates. Carbamates gained publicity after their inclusion in the Chemical Weapons Convention (CWC) list of Schedule 1 chemicals in 2019. According to the site of the Organisation for the Prohibition of Chemical Weapons (OPCW) Schedule 1 (https://www.opcw.org/chemical-weapons-convention/annexes/annex-chemicals/schedule-1, accessed on 17 December 2023), the listed carbamates included quaternaries and bisquaternaries of dimethylcarbamoyloxypyridines as depicted in Table 1. The purpose of this article is to provide a holistic view of the agents. In this regard, as will be outlined here, the list of exceedingly toxic carbamates is significantly more extensive and diverse. Thus, the agents included in the CWC list represent only a small fraction of the developed carbamate nerve agents (CNAs).

The rationale for the development of CNAs during the 1940s was based on the search for compounds for military use that were non-volatile, stable during storage, and lethal within a few minutes after exposure. In this regard, various compounds were considered prototypes including bacterial toxins, ricin, and physostigmine. The first two were not pursued further due to their delayed toxic action, while carbamate physostigmine was considered a promising lead compound due to its high toxicity and rapid onset of toxic symptoms [4].

As mentioned, CNAs have been marginally studied outside military literature. Three review articles have been published on CNAs (in 2013, 2022, and 2024), but these do not provide a holistic approach and are missing many potential agents [5,6,7]. Specifically, these articles do not include the mono-quaternized compounds that were first developed in the 1940s (some of these are exceedingly toxic and are labelled here as first-generation CNAs). Additionally, they did not attempt to provide a classification system based on their structure and their ability to act as incapacitants or lethal agents.

**Figure 1:**
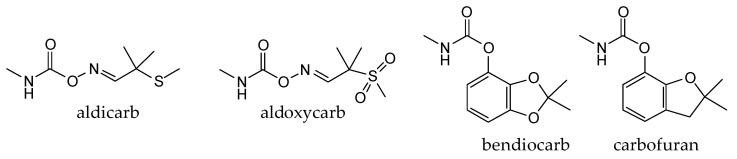
Representative examples of the most toxic carbamate pesticides [aldicarb LD_50_ rat oral: 650 μg∙kg^−1^; aldoxycarb LD_50_ rat oral: 20,000 μg∙kg^−1^, rat iv 14,900 μg∙kg^−1^; bendiocarb LD_50_ rat oral: 40,000 μg∙kg^−1^, mouse oral: 45,000 μg∙kg^−1^; carbofuran LD_50_ rat oral: 5000 μg∙kg^−1^, mouse iv: 450 μg∙kg^−1^; dog oral: 19,000 μg∙kg^−1^; mouse oral: 2000 μg∙kg^−1^] (data were retrieved from Pubchem, http://pubchem.ncbi.nlm.nih.gov, accessed on 21 September 2024).

At this point, it should be noted that carbamates both constitute an important class of pesticides and that many drugs are carbamate derivatives [5]. Certain carbamate pesticides, as shown in Figure 1, are very toxic compounds, especially aldicarb. As will be discussed in the following sections, they lack the characteristic moiety of CNAs that includes a quaternized nitrogen moiety either directly attached to, or spaced one carbon atom from, an aromatic ring. Further, although these pesticides exhibit high toxicity, this toxicity is significantly lower than that of the second-generation CNAs.

To date, CNAs have not been used in any military conflict or chemical terrorism act. This may be related to the fact that these agents are solids and therefore more sophisticated and complicated systems are required for their dissemination. Nevertheless, CNAs could in theory be used to contaminate water or in assassination attempts in a manner that resembles the previous use of VX and A-agents [8,9]. This calls for a categorization of the compounds as well as future experimentation to better understand their chemistry and toxicology, which are needed to develop effective countermeasures both for treatment of potential intoxications as well as for decontamination. In the present article, a comprehensive review of the developed CNAs has been performed and these compounds have been categorized for the first time to our knowledge based on their structure to establish their chemical and toxicological profile.

## 2. Methods

The subset of CNAs that were included in the CWC list of Schedule 1 chemicals, shown in Table 1, were obtained from the site of the OPCW (https://www.opcw.org/chemical-weapons-convention/annexes/annex-chemicals/schedule-1, accessed on 17 December 2023). The information on CNAs was obtained from Google Patents, PubMed, and Google. Keywords used for searching were “carbamate nerve agents”, “chemical warfare agents”, “EA-3990”, “TL-599”, and “toxic phosphorus compounds”. Only military reports, patents, and scientific papers were used for the construction of this article. Declassified documents on CNAs were also searched for in the Defense Technical Information Center (DTIC, Fort Belvoir, VA, USA).

## 3. Carbamate Nerve Agents (CNAs)

CNAs were initially separated into two categories that were named the first and second generation. The first generation of CNAs was developed in the 1940s, and the second generation during the 1970s and 1980s. More importantly, the second generation of CNAs includes agents that show more complicated chemical structures compared to the first generation. Further, the most toxic compounds of second-generation CNAs tend to have symmetric chemical structures. In the following paragraphs, their detailed structure and toxicity will be reported.

### 3.1. First Generation of CNAs

**Figure 2:**
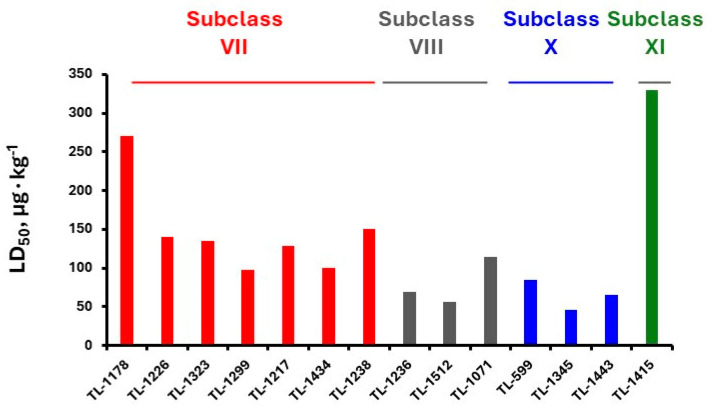
Toxicities of representative compounds from subclasses VII, VIII, X, and XI. The selected compounds exhibit LD_50_ (sc mice) lower than 500 μg∙kg^−1^. Thus, they may be considered potential CWAs. When a range of toxicity values is given for a compound, e.g., TL-1238 (125–175 μg∙kg^−1^) (in Table 2), the mean value has been added to generate the graph. As shown, the highest toxicity is exhibited by TL-1345.

The first generation of CNAs includes compounds possessing a benzene ring that carries one or more carbamate groups, and one or more quaternary ammonium groups (although in a certain subclass the ammonium group is replaced with sulfonium or arsonium). A total of 18 subclasses of first-generation CNAs have been reported and studied. The XIV–XVII subclasses contain molecules with more diverse substructures [4]. Many of these compounds are labelled with the abbreviation TL followed by a number, where TL stands for Toxicity Laboratory and indicates that toxicological studies have been carried out at the University of Chicago Toxicology Laboratory. As can be deduced from Table 2, only subclasses VII, VIII, X, and the compound TL-1415 from the XI subclass, are of military importance since the remaining compounds do not show high toxicity. The toxicity of certain first-generation CWAs is graphically represented in Figure 2.

### 3.2. Mechanism of Toxicity and Relationship with Structure

**Figure 3:**
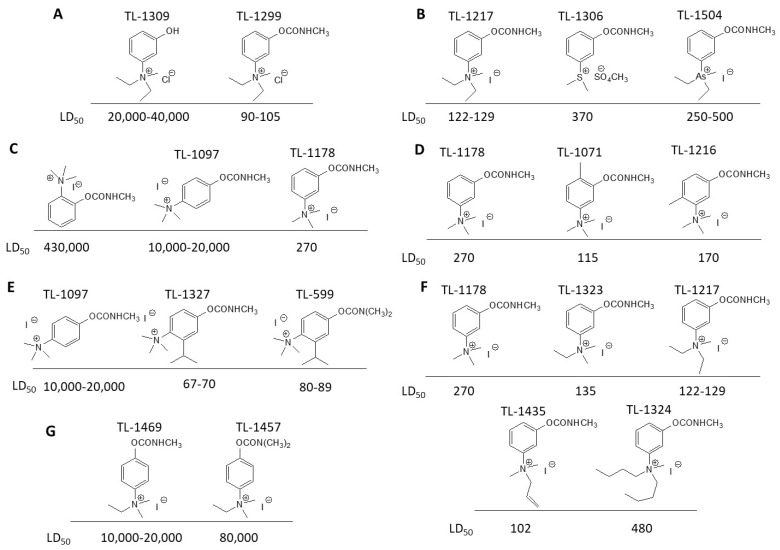
Correlation of structure with toxicity for first-generation CNAs. The LD_50_s are shown as μg∙kg^−1^ when administered as a water solution in mice (sc). TL-1309 is a control compound for class VII (lacks the carbamate moiety). (**A**–**G**) indicates various examples that compare carbamates and were used to extract the “toxicity rules”.

After synthesizing a large group of carbamates (327 compounds summarized in Table 2), certain rules that govern the relationship between structure and toxicity can be obtained [4], and can be summarized as follows:

1. The most toxic compounds contain both a carbamate and a quaternary ammonium salt, for example Figure 3A (comparison of TL-1309 with TL-1299). This is a universal observation and the replacement of carbamate with a hydroxy group or the deletion of the tertiary amine results in a loss of toxicity. Further, this became the basis for the generation of carbamates bearing quaternary ammonium group as CWAs;
2. The replacement of the ammonium group with sulfonium or arsonium slightly reduces the toxicity, for example Figure 3B (comparison of TL-1217 with TL-1306 and TL-1504). This rule has been derived from studying five compounds and no exceptions have been detected;
3. The introduction of the quaternary ammonium in the meta position relative to the carbamate group increases the toxicity of the compound (Figure 3C). In total, four pairs of compounds were examined;
4. In the compounds containing the quaternary ammonium in the meta position relative to carbamate, the further introduction of a methyl group at the ortho or para position relative to the carbamate further increases toxicity, for example Figure 3D. From a comparison of 14 pairs, 13 followed the rule and only 1 pair showed approximately equal toxicity with the addition of the methyl group;
5. The compounds that contain a quaternary ammonium in the para position relative to the carbamate can be converted to exceedingly toxic compounds by introducing an alkyl group (mainly isopropyl group) at the meta position relative to the carbamate (Figure 3E). This was deduced by studying nine compounds;
6. Changes in the methyl substituents of quaternary ammonium slightly increase the toxicity of the compounds (Figure 3F). However, the introduction of two bulky alkyl groups, e.g., two butyl groups like the agent TL-1324, results in a reduction in toxicity. This was based on studying two pairs of compounds;
7. N-methylcarbamates are usually, but not exclusively, more toxic than N,N-dimethylcarbamates (Figure 3G). Of 20 pairs examined, 14 followed this rule.

CNA toxicity is based on the potent inhibition of the enzyme acetylcholinesterase (AChE). Experimentally, it was shown that TL-1217 exhibited specificity for AChE relative to BuChE, with its toxic action being due to AChE inhibition in the peripheral nervous system. This is likely attributable to the positive charge of these compounds that prohibits them from penetrating the blood–brain barrier [11,12]. As a potential pretreatment, bis-pyridinium compounds have been tested and found to exhibit a protective effect against TL-1217 [13]. No other data are available, but it was hypothesized that treatments used in cases of carbamate pesticide intoxications would be appropriate for treating individuals exposed to CNAs [6].

### 3.3. Stability

The compounds are stable. When heated at 65 °C, they exhibit low decomposition after 2 months, indicating that at room temperature, they will remain stable for significantly longer periods. Thus, they may be prepared and stockpiled for future use. In alkaline or slightly acidic solutions, they rapidly decompose, and an acidic pH (<5) is required to inhibit their decomposition. As a precaution, it has been suggested to recrystallize the CNAs from solutions containing HCl. The less hygroscopic compounds are preferred for military applications. In this direction, the TL-1217 was selected as a potential CWA compared to TL-1299, which is highly hygroscopic [4].

Outside military literature, the compound T-1152 (or TL-1178) has been patented as rodenticide [14]. In this patent, it was disclosed that this compound is tasteless, which in combination with high toxicity makes it ideal for targeting mice and rats. Nonetheless, TL-1178 rapidly decomposes in slightly acidified water. Specifically, it was demonstrated that a 1:300,000 dilution of TL-1178 in slightly acidified water (pH 6.2) resulted in decomposition after half an hour, and an adjustment of the pH between 1 and 5 was necessary for maintaining its poisonous capacity for 48 h. For the preparation of the final formulation, the addition of tartaric acid was proposed. This hydrolysis results in the generation of isocyanates [15].

### 3.4. Synthesis of First-Generation CNAs

**Figure 4:**
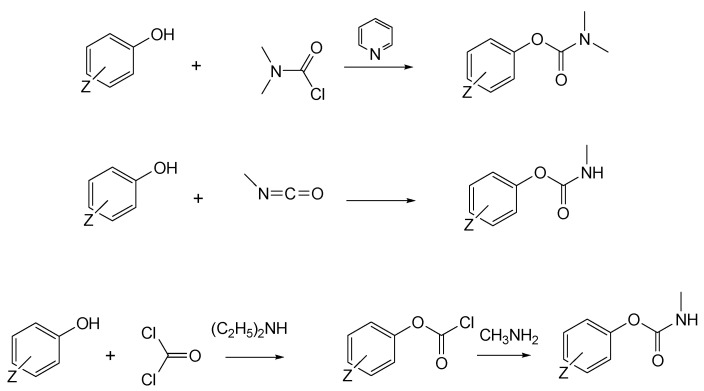
Reaction of phenols with dimethylcarbonyl chloride, methyl isocyanate, or phosgene followed by methylamine to generate the first generation of CNAs.

General synthesis involves the use of the appropriate phenol precursors and methyl isocyanate or dimethylcarbonyl chloride. Alternatively, the corresponding phenols can be converted to carbamates through a reaction with phosgene in the presence of diethylamine followed by methylamine, albeit at lower yields relative to the methyl isocyanate method (Figure 4). The conditions for the laboratory preparation of carbamates from the reaction between (o-, m-, p-) dimethylaminophenols and alkyl or phenyl isocyanates or phosgene have been described in detail in a study aiming to develop new miotic agents [15].

**Figure 5:**
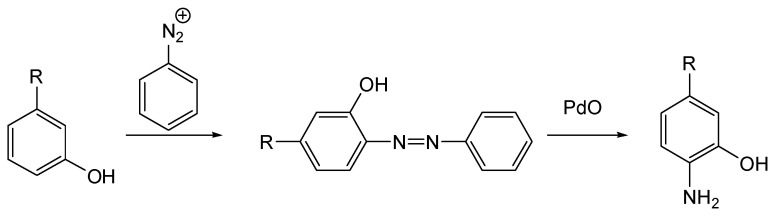
General synthesis scheme for p-amino-m-alkylphenols. These compounds are precursors of the first generation of CNAs.

The p-amino-m-alkylphenols can be prepared from m-alkylphenols through a reaction with diazotized aniline and the reduction of the resultant azo-compound in the presence of palladium oxide [16]. The respective scheme for the synthesis of the p-amino-m-alkylphenols is given in Figure 5.

Below, three representative examples for the synthesis of first-generation CNAs of military importance are given [4].

**Figure 6:**
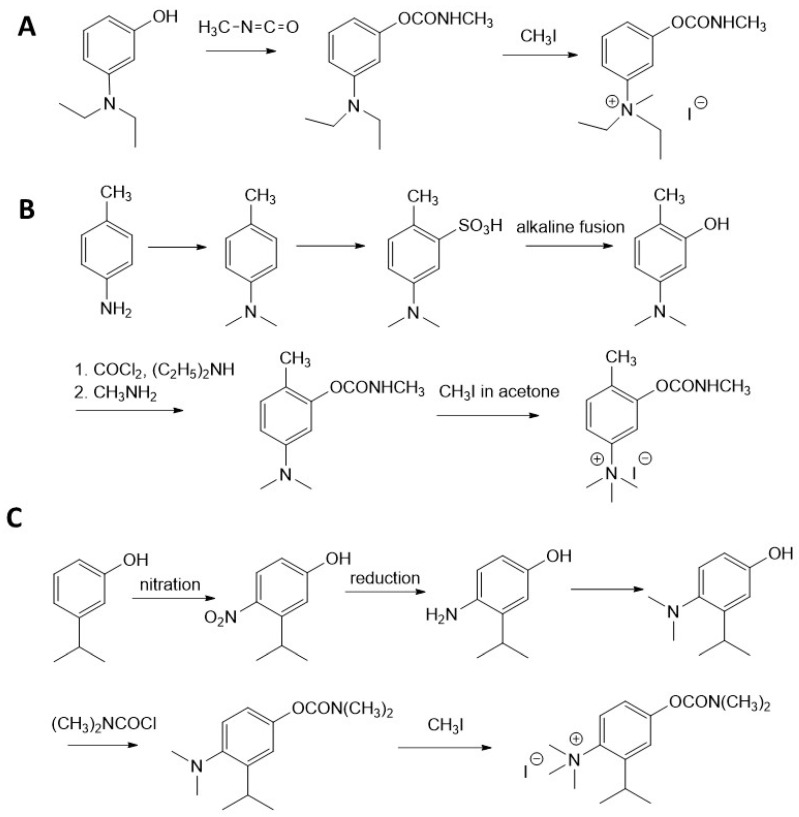
Examples of synthesis schemes for the first generation of CNA TL-1217 (**A**), TL-1071 (**B**), and TL-599 (**C**).

Production of TL-1217. The synthesis of TL-1217, which has been adapted for industrial production, begins with m-diethylaminophenol that reacts with methyl isocyanate. Both precursors are common industrial chemicals, as m-diethylaminophenol is used in the preparation of dyes and methyl isocyanate is used in the production of carbamate pesticides. The m-diethylaminophenyl N-methyl carbamate is produced and recovered in an 80% yield. This reacts with methyl iodide in acetone under reflux to yield the final product with yields of 79–86% (Figure 6A).

Production of TL-1071. The synthesis of TL-1071 is shown in Figure 6B. The upper part describes the synthesis of the precursor 2-methyl-5-dimethylamino-phenol from p-toluidine. The lower part shows the production of TL-1071 from this precursor. In a pilot plant, 39 lb of the compound were produced from the precursor with an overall yield of 39%.

Production of TL-599. This is one of the most toxic first-generation CNAs. The procedure for its synthesis is schematically shown in Figure 6C. It should be pointed out, however, that the starting material, i.e., m-isopropylphenol, is difficult to obtain, therefore making it unlikely to be clandestinely synthesized.

To this end, it should be noted that slight modifications in the above-mentioned examples could successfully yield the production of all other compounds mentioned in Table 1.

In summary, from a list of 327 chemicals (belonging to 18 different subclasses), it can be observed that these compounds are easy to synthesize, and certain compounds exhibit high toxicity, rendering them potential CWAs.

### 3.5. Second Generation of CNAs

During the 1970–1980s, a series of 28 patents were granted, describing the generation of a novel class of exceedingly toxic carbamates with many compounds exhibiting more than a 10-fold increase in toxicity relative to the first generation of CNAs. All these patents have been granted to the US government represented by the US Army. Subsequently, their chemical structure was visually inspected to identify potential key elements allowing their classification. All second-generation CNAs contain the dimethylcarbamoyl group and are solid quaternary ammonium salts. The key chemical characteristics that could allow for the classification of the compounds include the carbamate moieties that are attached to phenyl, pyridyl, mixed pyridyl-phenyl, isoquinolinium, or other groups. Additionally, the second generation of CNAs include mainly compounds that are bisquaternized (except class 14 which may be considered as precursors and class 4 that are quatro-quaternized).

Table 3 summarizes the classification of these CNAs based on the above-mentioned characteristics. Further, there are patents disclosing agents that are subcategories of other patents. These have been marked accordingly (Column entitled “comment”). Finally, there is the 25th subclass of agents that have been patented as potential incapacitants instead of lethal agents, since they display a high ratio of median effective dose (ED_50_)/LD_50_. The compound in subclass 19, also known as 3152CT, has been characterized in the published literature aside from the patents [17].

### 3.6. Toxicity

Based on the data shown in Table 3, certain structure–activity (toxicity) relationships can be deduced as follows:

1. Most compounds that have been investigated contain the two groups (carbamate and alkyl chain) at the ortho position on the aromatic ring. This most likely indicates that ortho substitution leads to the most potent compounds;
2. The introduction of a spacer containing 8–10 atoms between the two aromatic groups yields the compounds with the highest toxicity. This is directly deduced from studying the compounds that belong to subclass 3, within which, no exceptions were found;
3. For subclass 3, the highest toxicity is documented in compounds containing an eight-atom spacer (e.g., EA 3990). The total number of compounds that have been examined is 10;
4. It appears that the asymmetric compounds containing two quaternary nitrogens (subclasses 6–10, 12, 13, 15) exhibit lower toxicity compared to the respective symmetric compounds (subclasses 1–3). This is deduced by studying the three examples of subclass 1, the two examples of subclass 2, and the three examples from subclass 3 (that have an 8–10 atom spacer) with all the respective examples shown in subclasses 6–10, 12, 13, and 15;
5. In the asymmetric compounds, the spacer between the two quaternary nitrogens should consist of 10 atoms for the maximum toxicity (this is deduced from the comparison of the compounds shown in subclass 15). The finding may have been the reason for selecting the 10-atom spacer in subclasses 5–14 and 22;
6. The “half agents”, namely asymmetric agents of subclass 14 that can be considered precursors of full symmetric agents, show significantly reduced toxicity compared to their full analogs (compare EA 3887 from subclass 3 with the example compound shown in subclass 15);
7. Pyrimidinium-containing compounds are more toxic than their phenyl analogs (comparison of two respective pairs between subclasses 1 and 16 and two respective pairs between subclasses 2 and 17).

As mentioned previously and shown in Table 3, the agent that belongs to subclass 25 has a relatively low toxicity compared to other agents (LD_50_ iv mice 560 μg∙kg^−1^). However, it has ED_50_ (iv mice) 56 μg∙kg^−1^, which indicates that symptoms of poisoning appear in significantly lower amounts (10-fold) than are required to kill. Here, it must be pointed out that a difference between ED_50_ and LD_50_ of only 10-fold may not be enough to officially register it as an incapacitant. The most toxic compound appears to be EA 3990. This may be the reason why this agent was selected for field testing. Specifically, 0.7 pounds of the agent have been disseminated on Carroll Island, MD, USA without publishing the environmental and ecological effects [46]. In this report, EA-3990 is referred to as anticholinesterase. However, there are no studies that indicate the mechanism of action or the absorption, distribution, metabolism, and excretion (ADME) of these compounds. Given their positive charge, it is not likely that these compounds could easily penetrate the skin as is the case with organophosphate nerve agents. Previously, it was also speculated that the second generation of CNAs might also act through binding to acetylcholine receptors [6], something that needs to be clarified in future studies. To this end, it should be mentioned that the LD_50_ of VX is 14.5 μg∙kg^−1^ (iv mice) [47] in contrast to EA 3990 that exhibits 6.3 μg∙kg^−1^ for the same route of administration in mice, indicating the EA 3990 is more toxic that VX. Similarly, reviewing the data in Table 3, it can be observed that there are many carbamates more toxic than VX.

**Figure 7:**
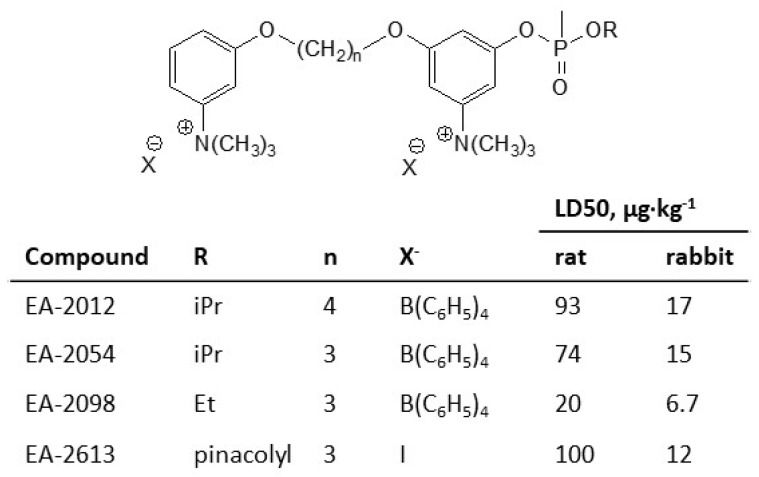
The phosphonate analogs of the subclasses 19 and 21 of CNAs. The toxicities are given for iv route.

Finally, it should be mentioned that there are also phosphonate analogs of the subclasses 19 and 21 (Figure 7), which are also exceedingly toxic compounds [48].

For the forensic detection of CNAs, mass spectrometry hyphenated methodologies have been suggested such as GC-MS, LC-MS, GC-MS/MS, and LC-MS/MS, where specific metabolites or degradation products could be monitored. More precisely, it has been speculated that CNAs could be subjected to Hofmann elimination (due to quaternized nitrogen) that in turn would produce valuable biomarkers for exposure to CNAs [7]. Nevertheless, this mechanism remains theoretical and has not been investigated either in vitro or in vivo. This highlights the need for thorough investigation of these “newly emerged” nerve agents. In addition, a new bioassay for the determination of potential exposure to the second-generation CNAs has been proposed based on their suggested ability to bind to acetylcholine receptors [7]. Again, whether these agents can bind to these receptors needs to be determined in the future and the proposed screening method for potential exposure remains speculative. Nevertheless, levels of blood cholinesterase activity could be used for an initial screening to identify potential exposure to nerve agents, as they have been used in cases of carbamate pesticide poisoning [49], where, however, it was not possible to discriminate between organophosphates and CNAs. Still, this would just be a preliminary screening since the compounds that inhibit AChE belong to many classes, and more advanced methods for analysis are required to confirm CNA exposure.

### 3.7. Synthesis

Details on the synthesis of these compounds are described in the relative patents. In general, their synthesis is relatively simple, with no requirement for complex reactions or equipment or extensive purification methods. This feature renders these compounds “ideal” for clandestine production. In general, the second-generation CNAs can be derived after synthesizing the aromatic ring containing carbamate moieties and cross-linking them with the appropriate linker.

**Figure 8:**
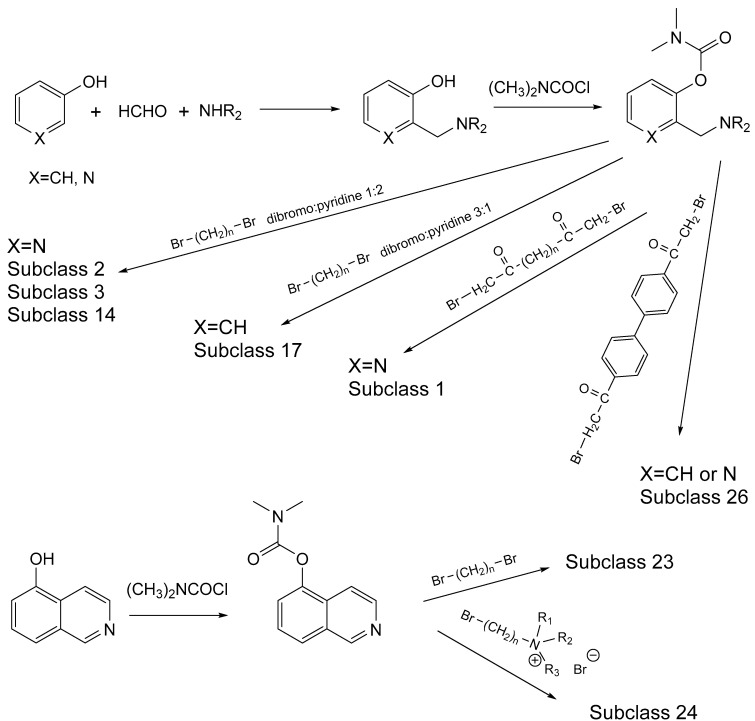
A synthesis scheme of selected compounds of the second generation of CNAs.

The most toxic derivatives contain the picolinyl group. The synthesis of the picolinyl derivatives with the Mannich reaction between 3-pyridol, formaldehyde, and the appropriate amine has been known since 1949 [50]. The Mannich reaction can also be applied to the introduction of the carbamate moiety in the phenyl group. Subsequently, these chemicals can react with the appropriate α, ω-dihalogen crosslinker to generate symmetric toxic agents as shown in Figure 8. The reaction with the crosslinker is straightforward and the product can be obtained simply by incubating the reagents at room temperature for one week. Alternatively, reflux for a few hours can be applied. The CNAs are recrystallized, filtered, treated with charcoal, and then filtered again.

The synthesis of the asymmetric compounds, e.g., subclasses 5–10, can be carried out by mixing the compound in subclass 14 with the appropriate amine. The reaction can be carried out in a solvent (acetonitrile, acetone, acetonitrile: chloroform, etc.) by incubation for more than a week at room temperature or by refluxing for 3–24 h. Ion exchange can be applied to exchange the anions, e.g., replacing the Br^−^ with B(C_6_H_5_)_4_^−^ [23].

Based on Figure 8, the isoquinoline derivatives are the easiest CNAs to be synthesized, since they require fewer steps starting from 5-hydroxyquinoline. Their synthesis only requires the reaction of 5-hydroxyquinoline with dimethyl carbamoyl chloride and then the reaction of the produced carbamate with α, ω-dibromoalkane. In conclusion, the second-generation CNAs include more complex agents that are straightforward to synthesize.

## 4. Nitrosocarbamates

**Figure 9:**
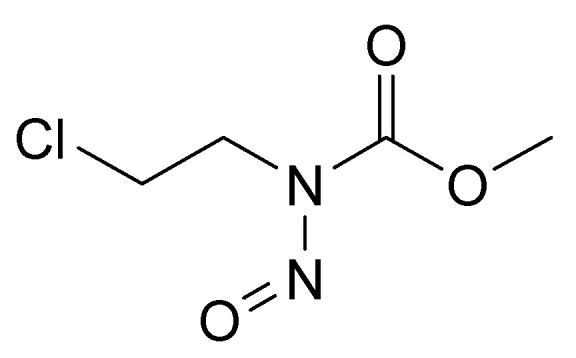
The structure of KB-16.

It should be noted that there is another class of potential chemical warfare agents, the nitrosocarbamates, with KB-16 (Figure 9) being the most important member [4,51]. However, as these compounds do not constitute nerve agents, but are vesicants, they will not be described here.

## 5. Conclusions

Here, a detailed presentation and classification of the synthesized CNAs has been carried out. CNAs came into the foreground after their inclusion as Schedule 1 chemicals in the CWC list. After analyzing the published literature and military files, we have identified a significantly large number of groups with potential CNAs that have not been discussed or summarized previously. Nevertheless, only two classes of carbamates were included in the CWC list. Recent research on CNAs is very limited, with only one study focusing on the development of a detection method [52] and three review articles that do not provide a holistic description and categorization of the compounds [5,6,7]. To investigate their chemical and biological properties, it is necessary to categorize them based on their structure. Such a classification has been performed here and should greatly advance the understanding of their properties and toxicities.

Finally, the simplicity in the chemical synthesis of CNAs renders them important threats for the population, e.g., during a terrorist act or in cases of intentional poisoning. Therefore, the study of their chemistry and toxicology is important, both for identifying their mechanism of action in detail, especially for the second-generation CNAs [7], and for developing new potential countermeasures including decontamination procedures.

## Figures and Tables

**Table 1:** The classes of CNAs included in CWC list of Schedule 1 chemicals.

Chemical (Schedule 1.16)	Example
1. Quaternaries of dimethylcarbamoyloxypyridines 1-[N,N-dialkyl(≤C10)-N-(n-(hydroxyl, cyano, acetoxy) alkyl (≤C10)) ammonio]-n-[N-(3-dimethylcarbamoxy-α-picolinyl)-N,N-dialkyl(≤C10) ammonio]decane dibromide (n = 1–8)	1-[N,N-dimethyl-N-(2-hydroxy)ethylammonio]-10-[N-(3-dimethylcarbamoxy-α-picolinyl)-N,N-dimethylammonio]decane dibromide (CAS no. 77104-62-2) 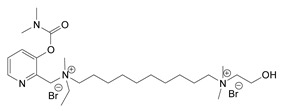
2. Bisquaternaries of dimethylcarbamoyloxypyridines 1,n-Bis[N-(3-dimethylcarbamoxy-α-picolyl)-N,N-dialkyl (≤C10) ammonio]-alkane-(2,(n-1)-dione) dibromide (n = 2–12)	1,10-Bis[N-(3-dimethylcarbamoxy-α-picolyl)-N-ethyl-N-methylammonio]decane-2,9-dione dibromide (CAS no. 77104-00-8) 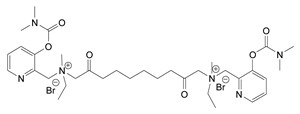

**Table 2:** Subclasses of the first generation of carbamate nerve agents (CNAs).

Subclass	General Formula	Category–Main Structural Characteristics				Representative Example(s)	LD_50_ (μg∙kg^−1^)	Number of Compounds Tested *	Notes	Potential as CWAs
		No of Benzene Rings Attached to Carbamate	No of Carbamate Groups	Position of Quaternary Ammonium Groups Attached to Benzene	Other Groups– Comments					
I	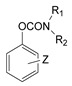	1	1	-	Could have various substituents	TL-997 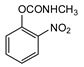	33,000 iv mice	13	All other tested compounds had LD_50_s > 40,000 (sc or iv)	-
II	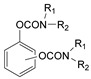	1	2	-	-	TL-978 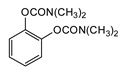	1400 sc mice (P)	7	All other tested compounds had LD_50_s > 10,000 (sc)	-
III	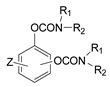	1	2	-	Has various substituents	TL-1160 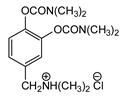	1000–5000 sc mice (W)	14	TL-1160, the most toxic analog has a quaternary ammonium, which is not present in other members	-
IV	Only TL-1115	1	3	-	-	TL-1115 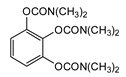	10,000–20,000 sc mice (W)	1		
V	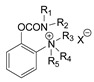	1	1	Ortho **	-	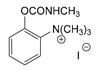	430,000 sc mice	2	All other tested compounds had LD_50_s > 80,000 sc	-
VI	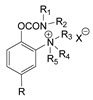	1	1	Ortho **	Has various alkyl substituents	SB-16 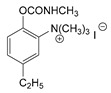	1250 sc mice	11		-
VII	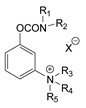	1	1	Meta **		T-1152 a.k.a. TL-1178 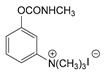	440 sc mice 270 sc mice (W) 260 sc rabbit 115 iv mice (W) 1000 sc dog (W)	42		++
						TL-1226 a.k.a. T-1690 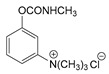	270 sc mice 140 sc mice (W) 70 iv mice (W)			
						TL-1323 a.k.a. T-1194 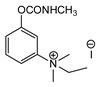	135 sc mice (W) 380 sc mice 130 sc rabbit			
						TL-1299 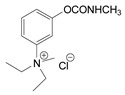	90–105 sc mice (W) 100–200 sc dog (W)			
						TL-1217 a.k.a. T-1123 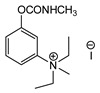	122–135 sc mice (W) 50–100 sc cat (W) 50 sc dog (W) 300–400 sc goat (W) 100–200 sc monkey (W) 100–200 sc sheep (W)			
						TL-1434 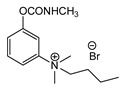	100 sc mice (W) 50–100 sc dog (W) 25–50 s.c. cat (W)			
						TL-1238 a.k.a. 3393 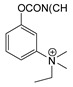	125–175 sc mice (W) 89 iv mice (W) 60 iv mice *** 400 sc rat (W) 100–200 sc cat (W) 300 sc dog (W)			
VIII	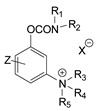	1	1	Meta **	Has various other substituents	TL-1236 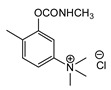	64–75 sc mice (W) 35 iv mice (W) 100 sc rat (W)	40		++
						TL-1512 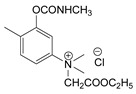	56 sc mice (W)			
						TL-1071 a.k.a. T-1708 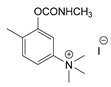	115 sc mice (W)			
IX	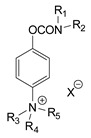	1	1	Para	-	TL-1456 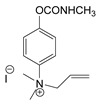	2500–5000 sc mice (W)	18	All other tested compounds had LD_50_s > 20,000	-
X	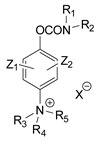	1	1	Para	Has various other substituents	TL-599 a.k.a. SB-8 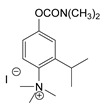	80–89 sc mice (W) 75 sc mice 200 sc rat (W) 168–265 ip mice (W) 100–200 sc dog (W) 200–300 sc cat (W)	73		+
						TL-1345 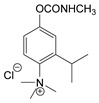	45–47 sc mice (W) 103 sc rat (W) 25–50 sc rabbit (W) 50–100 sc dog (W) 100 sc cat (W) 100–200 sc monkey (W)			
						TL-1443 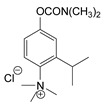	65 sc mice (W)			
XI	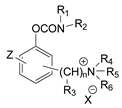	1	1	On an alkyl chain	-	TL-1415 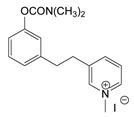	330 sc mice (W)	37	All other tested compounds had LD_50_s > 500	+/− only TL-1415
XII	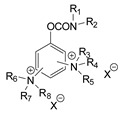 or 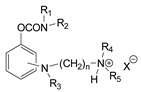	1	1	Variable positions		AR-27 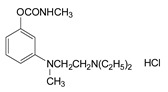	100 iv mice	8	All other tested compounds had LD_50_s > 7000	-
XIII	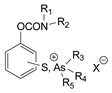	1	1		Sulfonium or arsonium	TL-1479 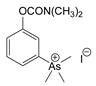	500–1000 sc mice (W)	5		-
XIV	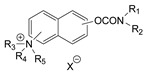		1	Variable position	Naphthalene carries the carbamate moieity	TL-1406 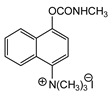	310 sc mice (W)	5	All other tested compounds had LD_50_s > 4000	-
XV	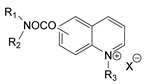 or 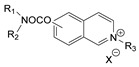		1	Variable position	Quinoline or isoquinoline group carries the carbamate moiety	T-1973 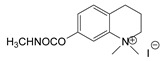	330 mice, no route was reported	11	All other tested compounds had LD_50_s > 500	-
XVI	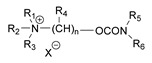				Carbamates with aliphatic alcohol derivatives	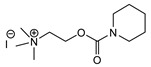	4000 sc mice	28	All other tested compounds had LD_50_s > 6250	-
XVII	Based on physostigmine				Physostigmines	TL-1380 (physostigmine salicylate) 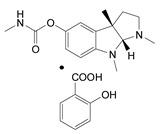	370 sc mice (W) 500 iv mice	7	All other tested compounds had LD_50_s > 750	
XVIII	variable				Carbamides and carbazates	AR-26 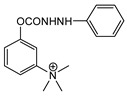	250 iv mice	5	All other tested compounds had LD_50_s > 80,000	

* The complete set of the compounds can be found in the relevant publication [4]. ** The position is reported relative to carbamate moiety. *** From [10]. (P) indicates that the agent was administered in paraffin oil. (W) indicates that the agent was administered in water. a.k.a. also known as. The ++ sign has been used to label the subclasses where more than 40% of the tested compounds exhibited LD_50_ lower than 500 μg∙kg^−1^. Thus, they have the potential to be used as chemical warfare agents (CWAs). The + sign indicates compounds that exhibit LD_50_ lower than 500 μg∙kg^−1^, which comprise a range of 10 to 39% of the total tested compounds. The +/− was used for the subclass XI since it included only one agent, TL-1415, that exhibits high toxicity.

**Table 3:** Chemical structure and toxicological properties of the second generation of CNAs.

Subclass	General Formula	Category *	Examples	LD_50_ iv (μg∙kg^−1^)		Patent	Comment	Ref	No of Compounds Tested	Covered by CWC
				Rabbits	Mice					
1	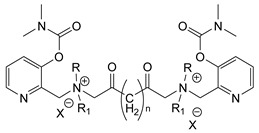	Pyridine (2)	R = R_1_ = Me, X = Br, n = 6	2.7	7	US4246416		[18]	18	2
			R = Me, R_1_ = Et, X = Br, n = 6	4	10					
			R = R_1_ = Me, X = Br, n = 4	2.7	10					
2	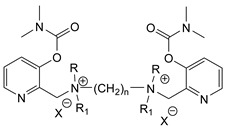	Pyridine (2)	R = Me, R_1_ = Et, X = Br, n = 10	4	11	US4686293		[19]	7	2
			R = Me, R_1_ = Et, X = Br, n = 8	4	6					
3	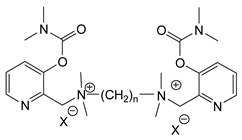	Pyridine (2)	X = Br, n = 3 (EA 4048)	>20,000	>32,000	US4677204	Sub-category of US4686293	[20]	10	2
			X = Br, n = 4 (EA 4038)	5600	3200					
			X = Br, n = 5 (EA 4026)	56	63					
			X = Br, n = 6 (EA 3948)	17.6	17.8					
			X = Br, n = 7 (EA 4181)	5.6	13					
			X = Br, n = 8 (EA 3990)	2.6	6.3					
			X = Br, n = 9 (EA 4056)	2.7	11					
			X = Br, n = 10 (EA 3887)	4.2	10					
			X = I, n = 10 (EA 3887A)	5	10					
			X = Br, n = 11 (EA 4057)	5	9					
4	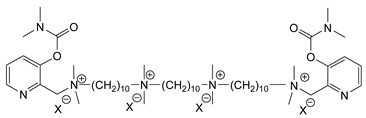	Pyridine (2)	X = B(C_6_H_5_)_4_ (tetraphenylboron)	8	32	US4672120		[21]	6	
5	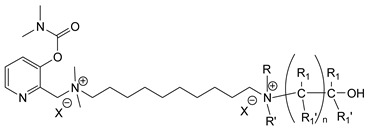	Pyridine (1)	R = R′ = Me, R_1_ = R_1_′ = R_2_ = R_2_′ = H, X = Br, n = 1	45	9	US4241212		[22]	15	1
			R = R′ = Me, R_1_ = R_1_′ = R_2_ = R_2_′ = H, X = Br, n = 2	56	36					
			R = Me, R_1_ = R_1_′ = R_2_ = R_2_′ = H, R′ = (CH_2_)_2_OH, X = B(C_6_H_5_)_4_, n = 1	54	14					
6	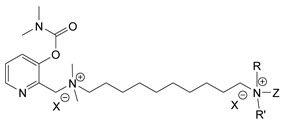	Pyridine (1)	R = R′ = Ζ = Butyl X = B(C_6_H_5_)_4_	5	28	US4672123		[23]	6	1
			R = R′ = Me, Z = Cyclohexyl X = Br	10	28					
7	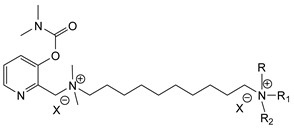	Pyridine (1)	R, R_1_, R_2_ form 3-quinuclidinol, X = Br	59	11	US4672119	Sub-category of US4672123	[24]	6	1
			R, R_1_ form pyrrolidine, R_2_ = Me, X = Br	6	10					
8	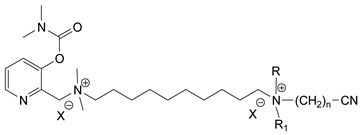	Pyridine (1)	R = R_1_ = Me, X = Br, n = 1	56	10	US4672124	Sub-category of US4672123	[25]	9	1
			R = R_1_ = Me, X = B(C_6_H_5_)_4_, n = 3	56	11					
9	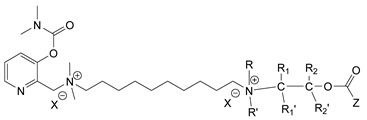	Pyridine (1)	R = R′ = R_2_ = Me, R_1_ = R_1_′ = R_2_′ = H, Z = Me, X = B(C_6_H_5_)_4_	6.3	13	US4672122 US4672069	Sub-category of US4672123	[26,27]	10	1
			R = R′ = Me, R_1_ = R_1_′ = R_2_ = R_2_′ = H, Z = Propyl X = Br	6	13					
10	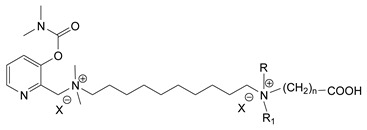	Pyridine (1)	R = R_1_ = Me, X = Br, n = 1	17	32	US4246418	Sub-category of US4672123	[28]	10	1
11	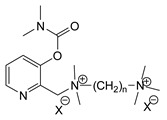	Pyridine (1)	X = Br, n = 10 (EA 3966)			US4675411	Sub-category of US4672123	[29]	15	1
12	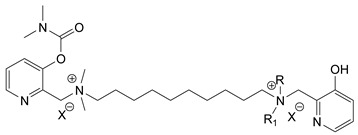	Pyridine (1)	R = R1 = Me X = B(C_6_H_5_)_4_	58	22	US4246415	As US4246416 but one carbamate	[30]	11	
13	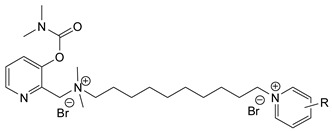	Pyridine (2)	R = H, X = Br	7	13	US4241210		[31]	7	
			R = p-CHNOH, X = B(C_6_H_5_)_4_	58	18					
14	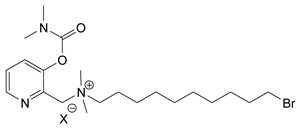	Pyridine (1)	X = Br	80	45	US4677205	Precursor of other carbamates, e.g., US4672119	[32]	6	
15	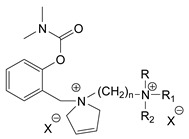	Benzene (1)	R = R_1_ = R_2_ = Me, X = B(C_6_H_5_)_4_, n = 10	6	13	US4240965		[33]	17	
			R = R_1_ = R_2_ = Me, X = B(C_6_H_5_)_4_, n = 8	46	14					
16	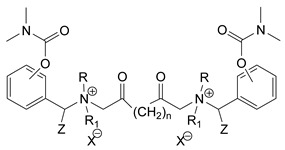	Benzene (2)	Ortho position carbamate, R = R_1_ = Me, Z = H, X = Br, n = 6	5	14	US4677222		[34]	23	
			Ortho position carbamate, R = R_1_ = Z = Me, X = Br, X = B(C_6_H_5_)_4_, n = 6	6	18					
			Ortho position carbamate, Z = H, X = Br, n = 4	5	56					
17	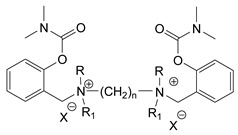	Benzene (2)	R = Me, R_1_ = Et, X = Br, n = 8	5	7	US H443	Sub-category of US4677222	[35]	13	
			R = Me, R_1_ = propyl X = Br, n = 10	10	45					
18	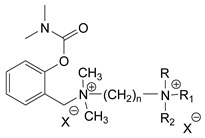	Benzene (1)	R = R_1_ = R_2_ = Me, X = Br, n = 10	7	22	US4241218		[36]	14	
			R = R_1_ = R_2_ = Me, X = B(C_6_H_5_)_4_, n = 8	7	14					
19	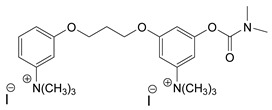	Benzene (2)				US3903135		[37]	1	
20	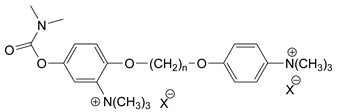	Benzene (2)	X = hydrogen oxalate, n = 3	50	44	US3919289		[38]	6	
21	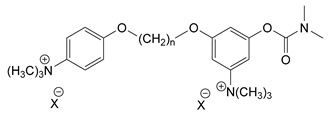	Benzene (2)	X = I, n = 2	7	16	US3901937		[39]	10	
22	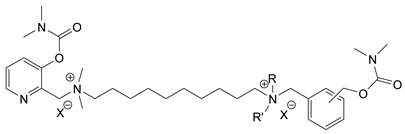	Mixed Pyridyl Benzyl	Para position carbamate, R = R’ = Me, X = B(C_6_H_5_)_4_	8	18	US4241211		[40]	12	
23	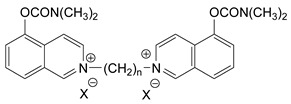	IsoQ	X = Br, n = 8	6	16	US4673745		[41]	19	
24	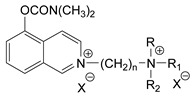	IsoQ **	R = R_1_ = R_2_ = Me, X = Br, n = 10	25		US4241209		[42]	16	
25	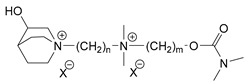	3-Q ***	X = Br, n = 10, m = 2		560 (MED 56)	US3956365 US3919241		[43,44]	15	
26	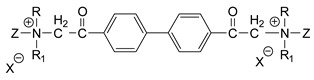	Phenyl or pyridine				US4692530		[45]	7	
	Examples: 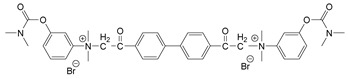			28	18					
	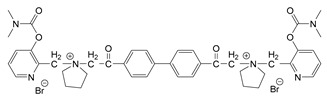			10	22					

* In parenthesis the number of the specific moieties is given. ** IsoQ: isoquinolinium. *** 3-Q: 3-quinuclidinol.

## Data Availability

The original contributions presented in this study are included in the article.

## References

[1] Pampalakis G., Kostoudi S. (2023). Chemical, physical, and toxicological properties of V-agents. Int. J. Mol. Sci..

[2] Opravil J., Pejchal J., Finger V., Korabecny J., Rozsypal T., Hrabinova M., Muckova L., Hepnarova V., Konecny J., Soukup O. (2023). A-agents, misleadingly known as “Novichoks”: A narrative review. Arch. Toxicol..

[3] Aroniadou-Anderjaska V., Apland J.P., Figueiredo T.H., De Araujo Furtado M., Braga M.F. (2020). Acetylcholinesterase inhibitors (nerve agents) as weapons of mass destruction: History, mechanisms of action, and medical countermeasures. Neuropharmacology.

[4] Chemical Warfare Agents, and Related Chemical Problems, Summary Technical Report of Division 9, National Defense Research Committee, Washington DC, 1946 (Unclassified). https://ia800107.us.archive.org/0/items/DTIC_AD0234270/DTIC_AD0234270.pdf.

[5] Voris D.G.R., Cavalcante S.F.A., Borges C.V.N., Lima A.L.S. (2024). Carbamates: Are they “Good” or “Bad Guys”?. J. Braz. Chem. Soc..

[6] Palermo G., Kovarik Z., Hotchkiss P.J. (2022). Newly scheduled carbamate compounds: A synopsis of their properties and development, and considerations for the scientific community. Toxicology.

[7] Ball J.C. (2013). Binding of quaternary ammonium salts to acetylcholine receptors: Possible chemical warfare nerve agents. Mil. Med. Sci. Lett..

[8] Nakagawa T., Tu A.T. (2018). Murders with VX: Aum Shinrikyo in Japan and the assassination of Kim Jong-Nam in Malaysia. Forensic Toxicol..

[9] Steindl D., Boehmerle W., Körner R., Praeger D., Haug M., Nee J., Schreiber A., Scheibe F., Demin K., Jacoby P. (2021). Novichok nerve agent poisoning. Lancet.

[10] Bülbring E., Chou T.C. (1947). The relative activity of prostigmine homologues and other substances as antagonists to tubocurarine. Brit. J. Pharmacol..

[11] Bajgar J., Patocka J. (1976). Anticholinesterase action of 3-diethylaminophenyl-N-methyl-carbamate methiodide in vitro and in vivo. Acta Biol. Med. Ger..

[12] Bajgar J., Patocka J. (1983). Inhibition of cholinesterase from different sources by 3-diethylaminophenyl-N-methylcarbamate methiodide in vitro. Sb. Ved. Pr. Lek. Fak. Karlov. Univ. Hradci Kral..

[13] Patocka J., Bajgar J. (1987). Protective effect of bis-pyridinium compounds on the rat brain acetylcholinesterase inhibition by carbamate in vitro. Biomed. Biochim. Acta.

[14] Aeschlimann J.A. (1932). Product for Destroying Animals. U.S. Patent.

[15] Stedman E. (1926). Studies on the relationship between chemical constitution and physiological action: Part I. Position isomerism in relation to the miotic activity of some synthetic urethanes. Biochem. J..

[16] Stevens J.R., Beutel R.H. (1941). Physostigmine substitutes. J. Am. Chem. Soc..

[17] Levin A.P., Jandorf B.J. (1955). Inactivation of cholinesterase by compounds related to neostigmine. J. Pharmacol. Exp. Ther..

[18] Sommer H.Z., Wicks G.E., Owens O.O. (1981). Chemical Agents. U.S Patent.

[19] Sommer H.Z., Owens O.O. (1987). Chemical Agents. U.S. Patent.

[20] Sommer H.Z., Krenzer J., Miller J.I. (1987). Chemical Agents. U.S. Patent.

[21] Sommer H.Z., Wicks G.E., Witten B. (1987). Chemical Agents. U.S. Patent.

[22] Sommer H.Z., Wicks G.E. (1980). Chemical Agents. U.S. Patent.

[23] Sommer H.Z., Wicks G.E. (1987). Chemical Agents. U.S. Patent.

[24] Sommer H.Z., Wicks G.E. (1987). Chemical Agents. U.S. Patent.

[25] Sommer H.Z., Wicks G.E. (1987). Chemical Agents. U.S. Patent.

[26] Sommer H.Z., Wicks G.E. (1987). Chemical Agents. U.S. Patent.

[27] Sommer H.Z., Wicks G.E. (1987). Chemical Agents. U.S. Patent.

[28] Sommer H.Z., Wicks G.E. (1981). Unsymmetrical Bis-Quaternary Amino Acids. U.S. Patent.

[29] Sommer H.Z., Miller J.I. (1987). Chemical Agents. U.S. Patent.

[30] Sommer H.Z., Wicks G.E. (1981). Picolyl Unsymmetrical Bis-Quaternary Carbamates. U.S. Patents.

[31] Sommer H.Z., Wicks G.E. (1980). Chemical Agents. U.S. Patent.

[32] Sommer H.Z., Wicks G.E. (1987). Chemical Agents. U.S. Patent.

[33] Sommer H.Z., Wicks G.E. (1980). Unsymmetrical Pyrrolino Benzyl Quaternary Compounds. U.S. Patent.

[34] Sommer H.Z., Wicks G.E., Owens O.O. (1987). Ketobenzylcarbamates. U.S. Patent.

[35] Sommer H.Z., Owens O.O. (1988). Chemical Agents. U.S. Patent.

[36] Sommer H.Z., Wicks G.E. (1980). Chemical Agents. U.S. Patent.

[37] Sommer H.Z. (1975). Method for Methylating and Quaternizing. U.S. Patent.

[38] Sommer H.Z., Krenzer J. (1975). Carbamates. U.S. Patent.

[39] Sommer H.Z., Krenzer J. (1975). Quaternary Carbamates. U.S. Patent.

[40] Sommer H.Z., Wicks G.E. (1980). Chemical Agents. U.S. Patent.

[41] Sommer H.Z., Owens O.O., Miller J.I. (1987). Isoquinilinium Chemical Agents. U.S. Patent.

[42] Sommer H.Z., Wicks G.E. (1980). Chemical Agents. U.S. Patent.

[43] Sommer H.Z., Miller J.I. (1976). Haloalkyl-Carbamoxyalkyl Derivates. U.S. Patent.

[44] Sommer H.Z., Miller J.I. (1975). Hydroxyquinuclidine Derivatives. U.S. Patent.

[45] Sommer H.Z. (1987). Chemical Agents. U.S. Patent.

[46] Ward F.P., Pinkham C.F.A. (1973). An Analysis of Chemical Agent Tests at Carroll Island, Maryland, in Recent Years.

[47] Bajgar J. (2012). Organophosphates and Nerve agents (Chapter 4). Nerve Agents Poisoning and Its Treatment in Schematic Figures and Tables.

[48] Natarelli G.E., Pinto F.G., Miller J.I. (1975). Toxic Phosphorus Compounds. U.S. Patent.

[49] Kumar S., Baggi T.R., Zughaibi T. (2022). Forensic toxicological and analytical aspects of carbamate poisoning-A review. J. Forensic Leg. Med..

[50] Stempel A., Buzzi E.C. (1949). 3-pyridols in the Mannich reaction. J. Am. Chem. Soc..

[51] Thompson R.H. (1947). The action of chemical vescicants on cholinesterase. J. Physiol..

[52] Zhang Q., Lv J., Xia J., Wang L., Qu G., Yang Y., Yang Y., Liu S. (2023). Rapid detection of carbamate nerve agent analogues using dually functionalized gold nanoclusters. Anal. Bioanal. Chem..

